# Silicon Nanowires Length and Numbers Dependence on Sensitivity of the Field-Effect Transistor Sensor for Hepatitis B Virus Surface Antigen Detection

**DOI:** 10.3390/bios12020115

**Published:** 2022-02-12

**Authors:** Chi-Chang Wu

**Affiliations:** Department of Electronic Engineering, National Chin-Yi University of Technology, Taichung 411030, Taiwan; ccwu@ncut.edu.tw

**Keywords:** silicon nanowire, NWFET, Hepatitis B surface antigen, HBsAg, sensitivity

## Abstract

Silicon nanowire field effect transistor (NWFET) sensors have been demonstrated to have high sensitivity, are label free, and offer specific detection. This study explored the effect of nanowire dimensions on sensors’ sensitivity. We used sidewall spacer etching to fabricate polycrystalline silicon NWFET sensors. This method does not require expensive nanoscale exposure systems and reduces fabrication costs. We designed transistor sensors with nanowires of various lengths and numbers. Hepatitis B surface antigen (HBsAg) was used as the sensing target to explore the relationships of nanowire length and number with biomolecule detection. The experimental results revealed that the sensor with a 3 µm nanowire exhibited high sensitivity in detecting low concentrations of HBsAg. However, the sensor reached saturation when the biomolecule concentration exceeded 800 fg/mL. Sensors with 1.6 and 5 µm nanowires exhibited favorable linear sensing ranges at concentrations from 800 ag/mL to 800 pg/mL. The results regarding the number of nanowires revealed that the use of few nanowires in transistor sensors increases sensitivity. The results demonstrate the effects of nanowire dimensions on the silicon NWFET biosensors.

## 1. Introduction

Interdisciplinary integration is a major research direction, and the integration of the fields of electronics and biomedicine can be highly beneficial. Biosensors are a research direction in biomedical electronics [1]. Silicon nanowire field effect transistor (NWFET) sensors, unlike traditional biomedical detection methods, have high sensitivity, are label free and compact, and provide real-time sensing [2,3]. The NWFET sensing method involves the use of nanowires connected to biological probes. Then, microchannel systems are used to inject solutions to be detected. Because biological probes have specificity to biological targets in the solution, when the probe captures the target, the charge carried by the target affects the carrier density of the field effect transistor channel and changes the current. Thus, the degree of change in the current can be used to detect the biomolecular target. The sensitivity of NWFET sensors can be increased by using small silicon nanowires, which increase the ratio of surface area to volume, thereby increasing sensitivity to the external environment. Numerous studies have fabricated nanoscale materials such as nanowires [4,5,6], nanotubes [7,8], and nanobelts [9,10] and applied them to biomedical sensors to increase their sensitivity.

Generally, two types of methods are used to fabricate nanowires. The first is the bottom-up method, in which metals are catalyzed to grow nanowires [11]. Although this method is low cost, the metal pollutants and nanowire control during the fabrication process are major problems [12]. The second type is the top-down method, in which photolithography and etching equipment for semiconductor manufacturing are used to define the dimensions and shape of the materials [13]. This process ensures control of the position of the nanowires, thereby facilitating mass production of nanowires of consistent quality [14,15]. However, because of the small size of nanowires, advanced photolithography equipment is required, which increases the fabrication costs [16]. To resolve the problems in these two methods, we used sidewall spacer etching to fabricate a silicon NWFET. Unlike the traditional top-down process, the proposed method does not require expensive nanoscale photolithography equipment and thus can reduce the cost of fabrication. In addition, the proposed method enables self-alignment, which reduces misalignment-induced error.

In research on biomedical sensors, increasing sensitivity is a crucial focus [17,18]. Studies have proposed several methods for increasing the sensitivity of NWFET sensors. For example, studies have used polyethylene glycol polymers to cover the surface of nanowires and prevent ion interference [19], employed plasma treatment to increase the density of biografting [20,21], and adjusted the surface treatment process to increase the probability that the target is grasped [22]. The present study explored the effect of nanowire design on sensitivity by designing NWFET sensors of various sizes. The number and length of the nanowires were changed to investigate their effects on biomolecule-sensing sensitivity. The research team of Park revealed the relationship between nanowire width and pH sensing sensitivity [23]; however, they did not investigate the effects of the length and number of nanowires on sensing sensitivity. The sensitivity of the NWFET is dependent on the electrical properties of the transistor. Generally, the sensitivity increases upon increasing the nanowire surface to volume (S/V) ratio. Moreover, the binding sites of the sensors and collision probabilities of the target molecules also influence the sensitivity. The length and number of nanowires are related to a transistor’s subthreshold swing (SS), S/V ratio, and biological binding sites. Variation in these two parameters affects sensor sensitivity; thus, the results of this study demonstrate the effects of nanowire size on sensing sensitivity.

This study used hepatitis B virus surface antigen (HBsAg) as the sensing target. HBsAg is a major biomarker of hepatitis B virus (HBV) that exists on the surface of the virus [24]. Cancer ranks as second among the world’s ten leading causes of death, and liver cancer consistently accounts for a high proportion of cancer diagnoses [25,26]. In Taiwan, the number of deaths caused by liver cancer is the highest or second highest among all deaths caused by cancers [27,28]. A main factor causing liver cancer is HBV infection, and approximately 400 million people worldwide have the virus [29,30]. The main route of transmission is through blood or body fluids. When blood carrying HBV enters the body, the person becomes infected with hepatitis B. Because the liver does not have nerves, those with HBV infection do not notice any symptoms in the initial stages. By the time the body begins to sends warning signals, the optimal treatment period has already passed [31,32]. Although medication can be used to treat hepatitis B, the recovery rate is low. The most commonly used diagnostic method for HBV is the enzyme-linked immunosorbent assay. However, this method is complex, requires a large number of samples, and has inadequate sensitivity [33]. Thus, this study used polycrystalline silicon (poly-Si) NWFET sensors with nanowires of various sizes to detect HBV. The results can assist in the diagnosis and treatment of hepatitis B.

## 2. Materials and Methods

### 2.1. Materials

HBsAg (Human), HBV surface antibody (HBsAb), and P16^INK4A^ antigen (Human) were purchased from Proteintech Inc. (Rosemont, IL, USA). The antigens and antibodies were stored in −20 °C environment after dilution. (3-aminopropyl)triethoxysilane [APTES; H_2_N(CH_2_)_3_Si(OC_2_H_5_)_3_, MW: 221.37 g/mol], analytical-grade ethanol (C_2_H_5_OH, absolute ≥99.8%), glutaraldehyde [GA; OHC(CH_2_)_3_CHO; MW: 100.12 g/mol], Bis-tris propane {BTP; CH_2_[CH_2_NHC(CH_2_OH)_3_]_2_, molecular weight (MW): 282.34 g/mol}, sodium cyanoborohydride (NaBH_3_CN; 95%, MW: 62.84 g/mol), and Tris hydrochloride [Tris HCl, (HOCH_2_)_3_CNH_2_•HCl, MW:157.6] were obtained from Sigma-Aldrich (St. Louis, MO, USA).

### 2.2. Fabrication of Poly-Si NWFET Devices

**Figure 1:**
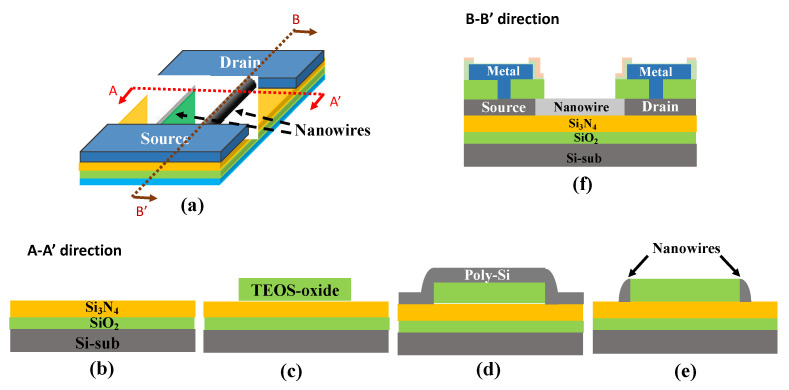
(**a**) Schematic representation of the poly-Si NWFET. (**b**–**e**) Flowchart of the nanowire fabrication process in the A-A’ direction. (**f**) Cross-sectional structure of the poly-Si NWFET in the B-B’ direction.

The poly-Si NWFETs were fabricated in the Taiwan Semiconductor Research Institute, Hsinchu, Taiwan. This study used sidewall spacer etching to fabricate the poly-Si nanowires. Figure 1a shows a schematic representation of the poly-Si NWFET, and Figure 1b–e presents a flowchart of the nanowire fabrication process. First, a 100 nm SiO_2_ layer, a 50 nm Si_3_N_4_ layer, and a 100 nm tetraethyl orthosilicate (TEOS) oxide layer were deposited on a silicon wafer substrate in that order (Figure 1b). A photo mask was used to define patterns and etch the TEOS oxide. Then, a dummy gate was fabricated (Figure 1c). Next, a 100 nm amorphous silicon layer was deposited. Through a long-duration, low-temperature thermal annealing process, the amorphous silicon was converted to polycrystalline silicon (Figure 1d). Next, a photo mask was used to define patterns, and ion implantation was used to define the source and drain areas. Subsequently, dry etching was performed to remove the polycrystalline silicon layer. With the etching time properly controlled, the sidewall spacer etching technique naturally leaves polycrystalline silicon on both sides of the dummy gate and forms the poly-Si nanowires (Figure 1e). The formation of nanowires is determined by the length and number of dummy gates. Therefore, we used dummy gates of various sizes to fabricate the poly-Si NWFETs. Then, a layer of TEOS oxide was deposited, the pattern was defined, and the contact hole of the source and drain electrodes were etched before metal deposition. Through etching, the position of the metal pad was defined. Finally, SiO_2_–Si_3_N_4_ was deposited to protect the transistor. Etch back was used to expose the detection region for subsequent biomedical sensing. The cross-sectional structure of the poly-Si NWFET in B-B’ direction is presented in Figure 1f. To prevent the detection region from being polluted or oxidized, a layer of photoresist was used to protect the chip. The photoresist layer was removed and the detection region was cleansed just before biomedical sensing. All fabricated chips were stored in a nitrogen environment.

### 2.3. Chemical Modification of the Sensor Chip Surface

The surface of the fabricated poly-Si NWFET sensors required chemical modification to fix biomolecules onto the nanowire surfaces and facilitate biological sensing. Before the surface chemical modification, the photoresist covering on the chips was removed through soaking in a photoresist remover (EKC 830) at 90 °C for 15 min. Then, the chips were cleaned and blow-dried. Next, the chips’ surfaces underwent oxygen plasma treatment to form OH¯ terminal on the nanowire surfaces. Then, the chips were soaked in a diluted (3-aminopropyl)triethoxysilane APTES solution (volume ratio of APTES to ethanol, 1:49) to facilitate a chemical reaction for 30 min. After the chips were dealcoholized, the NH_2_ surface modification was complete. Deionized water was then used to wash the chips before heating and drying. After the chips were cooled, they were soaked in a diluted GA solution (ratio of GA to BTP, 1:19) to facilitate a chemical reaction for 1 h and to finalize the aldehyde modification on the nanowire surfaces, which facilitated the bonding of aldehyde at the nanowire end-point with biomolecules.

### 2.4. HBsAb Biografting

**Figure 2:**
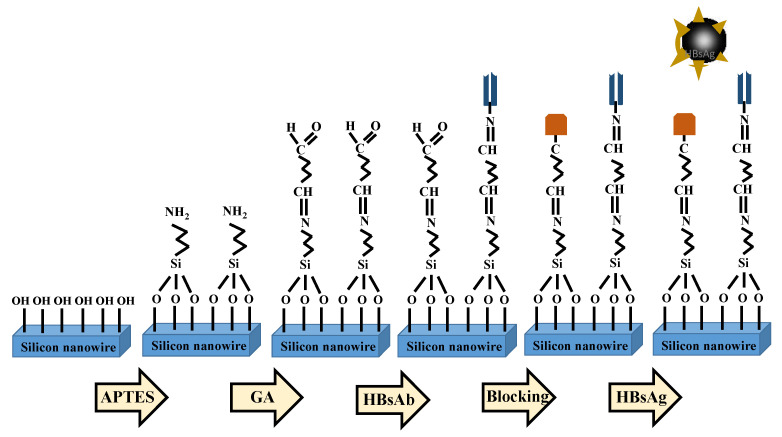
NWFET sensor surface chemical modification and biografting.

After chemical modification of the nanowire surfaces, HBsAb at a concentration of 1 μg/mL was dripped onto the nanowires and left to stand for 12 h to enable bonding between HBsAb and the surface aldehyde. Then, BTP was used to rinse off unbonded HBsAb. The unreacted aldehyde terminals on the sensor surface were then blocked by a 4 mM solution of NaBH_3_CN (volume ratio of NaBH_3_CN to Tris HCl, 1:249) for 30 min. The addition of NaBH_3_CN solution on the nanowire surface not only caused a complete reduction in the labile Schiff base intermediate between the aldehyde and amino terminals, but also transformed the unreacted aldehyde terminals to chemically stable bonds, preventing false positive detection results in the subsequent HBsAg sensing. After the BTP solution was used to rinse the chips, nitrogen was used to blow-dry the chips. Then, biological HBsAg detection was conducted. Figure 2 presents the surface chemical modification and biografting processes.

## 3. Results and Discussions

**Figure 3:**
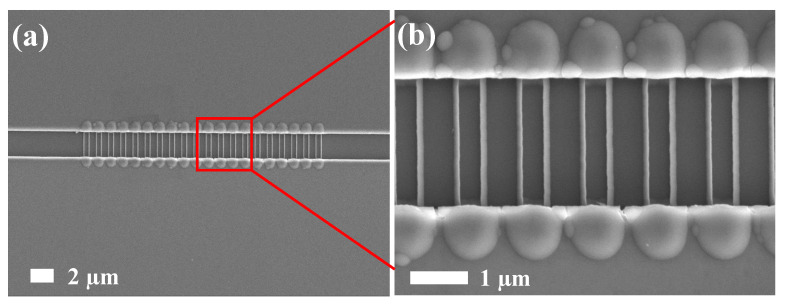
FESEM top-view images of the fabricated poly-Si nanowires. (**a**) Global-view image. (**b**) Enlarged image of the nanowires.

Nanowire morphology and size were determined through field-emission scanning electron microscopy (FESEM; JOEL, FESEM JSM-6700F). Figure 3 presents top-view FESEM images, indicating that 40 nanowires formed. Because nanowires formed on both sides of the dummy gate and because one dummy gate could help form two nanowires, the number of dummy gates was manipulated to control the number of nanowires formed. The length and width of the nanowires were 1.6 µm and approximately 100 nm, respectively (Figure 3).

**Figure 4:**
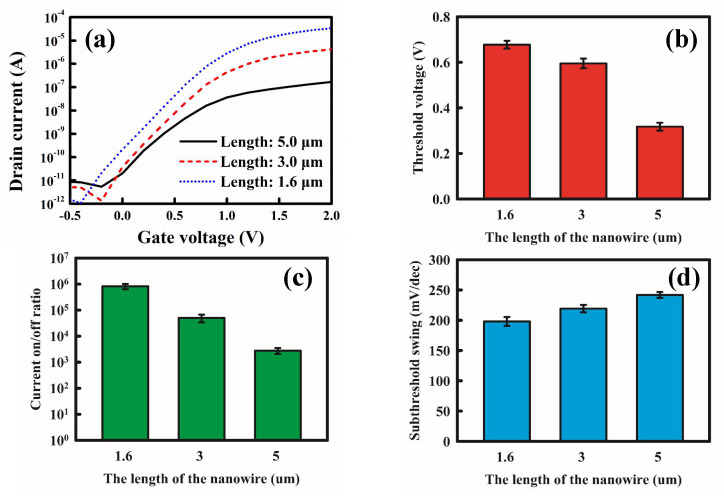
Transistors with nanowires of various lengths. (**a**) *I*_*D*_–*V*_*G*_ curves. (**b**) Threshold voltages. (**c**) ON/OFF current ratios. (**d**) SS.

Before biomedical sensing, we determined the basic characteristics of the poly-Si NWFETs by analyzing the component current–voltage curves. The drain operating voltage was 0.5 V, and we increased the gate voltage from −1 to 2 V. The characteristic curves of the drain current versus the gate voltage (*I*_*D*_–*V*_*G*_) of the transistors of various sizes were measured to determine the effects of the length (1.6, 3, and 5 µm) and quantity (10, 20, and 40 nanowires) of the nanowires on the basic electrical properties of the transistors. Figure 4 presents the basic electrical properties of the various NWFETs, and Figure 4a presents their *I_D_*–*V*_*G*_ characteristic curves. The curves indicate that short nanowires resulted in large ON currents in the transistors and increased the slope of the current in the subthreshold region. The current equation of the field effect transistor at the saturation region is as follows [34]:

(1) $$I_{D}=m\;\mu_{eff}\;C_{ox}\frac{W}{L}\;{\left(V_{G}-V_{TH}\right)}^{2}$$

where *m* is a constant which is related to the doping density; *μ_eff_* is the mobility of carriers, *C_ox_* is the gate oxide capacitance per unit area, *W* is the channel width, and *L* is the channel length. V_TH_ is the threshold voltage of the transistor.

According to Equation (1), *I*_*D*_ is inversely proportional to *L*. In a nanowire transistor, the nanowire length is the channel length. Moreover, parasitic resistance of the nanowire also affects drain current. Therefore, drain current decreases upon increasing nanowire length. The formula is consistent with the measured results.

By finding the square roots of the variables on both sides of Equation (1), we obtained the linear relationship between the *I*_*D*_^1/2^ and (*V*_*G*_−*V*_*TH*_) of the transistors in the saturation region. The line was then extrapolated to the X axis to extract the *V*_*TH*_ of the transistor. Figure 4b presents the *V*_*TH*_ of the transistors with the nanowires of various lengths in a histogram. The results indicate that as nanowire length increased, *V*_*TH*_ decreased slightly. Figure 4c presents the ON/OFF current ratios for the various transistors. The ON current was determined for the *I*_*D*_ value when *V*_*G*_ = 2 V, whereas the OFF current was the lowest when *V*_*G*_ = −0.5–2 V. Because the ON current increased considerably upon decreasing the nanowire length, whereas the OFF current varied only slightly, the ON/OFF current ratio of the transistor with 1.6 μm long nanowires was approximately 10 ^2^-fold higher than that of the transistor with 5 μm long nanowires.

The *I*_*D*_–*V*_*G*_ plot also displays the SS of the transistors in the subthreshold region, which represents the ability of an applied gate voltage to control the drain current. The formula is as follows:

(2) $$\mathrm{SS}=\frac{\partial V_{G}}{\partial \left(logI_{D}\right)}$$

According to Equation (2), SS is the inverse of the slope of a transistor’s *I*_*D*_–*V*_*G*_ curve. When the slope of the transistors in the subthreshold region increased, SS decreased, and the control capacity of transistor’s *V_G_* over *I*_*D*_ was strengthened. Generally, the shorter the channel length of the transistor, the lower the SS that is obtained. Figure 4d presents the SS of the various transistors. The results reveal that as nanowire length decreased, SS decreased, and the electrical properties of the transistors were enhanced. Short nanowires improved the transistor’s performance (Figure 4a–d).

**Figure 5:**
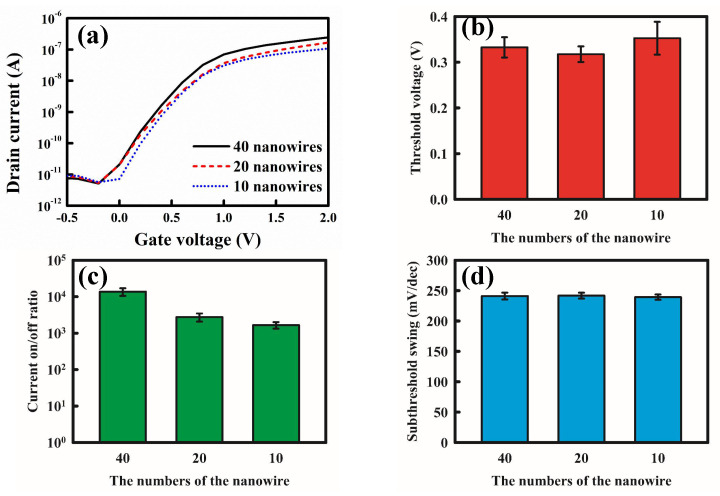
Characteristics of NWFETs with various numbers of nanowires. (**a**) *I*_*D*_–*V*_*G*_ curves. (**b**) Threshold voltages. (**c**) ON/OFF current ratios. (**d**) SS.

Figure 5 displays the basic electrical properties of the transistors with various numbers of nanowires. Figure 5a presents the *I*_*D*_–*V*_*G*_ characteristic curves of the transistors with 10, 20, and 40 nanowires. As the number of the nanowires increased, the ON current increased because the number of channels for electron flow, hence current, increased. Increasing the number of the nanowires can be regarded as increasing the channel width, and according to Equation (1), when channel width *W* increases, *I*_*D*_ increases. Figure 5b–d presents the threshold voltages, ON/OFF current ratio, and SS, respectively. The results indicate that as the number of nanowires increased, the ON/OFF current ratio increased. However, the number of nanowires had little effect on threshold voltage and SS.

**Figure 6:**
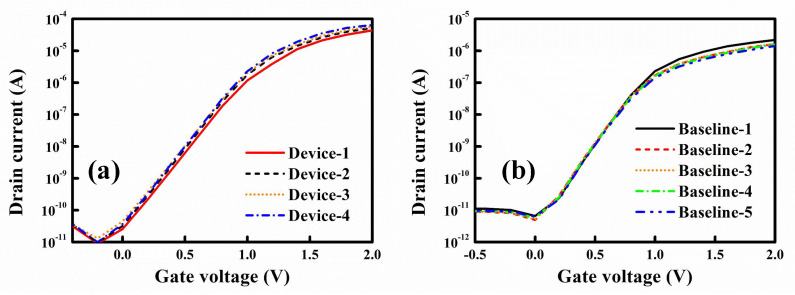
Stability of poly-Si NWFET devices. (**a**) *I*_*D*_–*V*_*G*_ curves of four NWFET devices with different nanowires. (**b**) Baseline *I*_*D*_–*V*_*G*_ curves of NWFET devices measured five times.

Stability is a crucial factor for biomedical sensors. A sensor with low stability can easily generate false positives or negatives. Figure 6a presents the *I*_*D*_–*V*_*G*_ characteristic curves of four randomly measured NWFET devices. The curves are similar, indicating that the fabricated poly-Si NWFET chips had favorable stability. Figure 6b presents the *I*_*D*_–*V*_*G*_ curves of a sensor measured in the buffer before the addition of the biological target. The curves are the baselines and were measured every 30 sec for a total of five times. The five *I*_*D*_–*V*_*G*_ curves almost overlap, indicating the high stability of the NWFET sensors (Figure 6b). The buffer did not affect detection.

**Figure 7:**
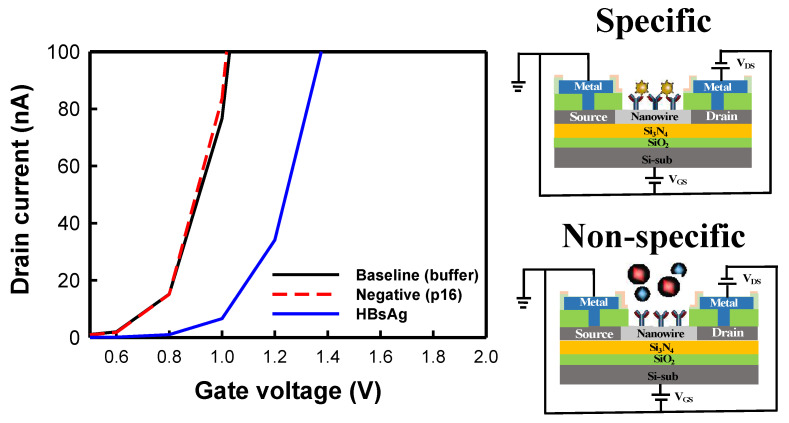
Specific detection results of poly-Si NWFET biosensors. The negative control sample used in this study was p16 antigen.

Specific detection is a critical indicator for assessing biomedical sensors. We used NWFET devices with 40 nanowires of 5 µm in length to test the specific detection of HBsAg. After HBsAb was bonded to the nanowire surfaces and blocking was performed, the nanowires were placed in 10 mM buffer to measure the baseline *I*_*D*_–*V*_*G*_ curves. Then, a key protein of cervical cancer p16 was injected at a concentration of 1 pg/mL. Because p16 does not react to or bond with HBsAb, this group was used as the negative control group. After 30 min, buffer was used to rinse the chips. The *I*_*D*_–*V*_*G*_ curves of the negative control group were obtained by measuring the sensor electrical properties. Finally, HBsAg was added at a concentration of 80 fg/mL and left to stand for 30 min before rinsing to remove unbonded antigens from the nanowires. Subsequently, the *I*_*D*_–*V*_*G*_ curves were measured. Figure 7 presents the specific detection results of the poly-Si NWFET sensors. The results revealed that in the negative control test, the added p16 antigen did not correspond to HBsAb and that the two did not bond. Thus, in the rinsing process, the p16 antigen was removed, and the measured *I*_*D*_–*V*_*G*_ curves overlapped with the baseline curves. When HBsAg was added, it reacted to and bonded with the antibody. The negative charge on HBsAg caused the curve to shift rightward. The results revealed that the NWFET sensors exhibited high specificity to HBsAg detection.

We used the poly-Si NWFET sensors to detect HBsAg and calculated their normalized voltage shift when detecting targets at various concentrations to identify the effects of the length and number of nanowires on HBsAg detection. Normalized voltage shifts were calculated using the drain current corresponding to the baseline *V*_*TH*_ value (*V*_*TH,Baseline*_) of the NWFET sensor as the baseline current, which was then substituted into the curves for the various concentrations to obtain the detected *V*_*TH*_ value (*V*_*TH,HBsAg*_). The equation is as follows:

(3) $$\mathrm{Normalized}\;\mathrm{voltage}\;\mathrm{shift}=\frac{\left(V_{TH,HBsAg}-V_{TH,Baseline}\right)}{V_{TH,Baseline}}$$

**Figure 8:**
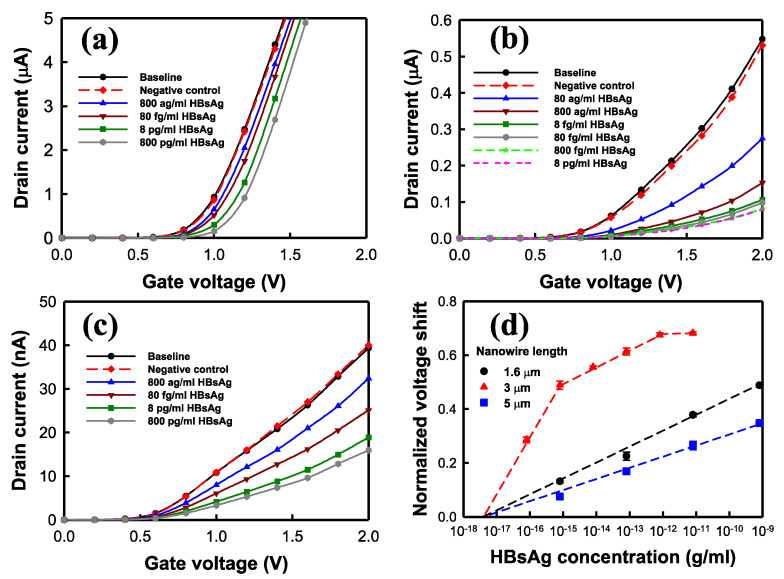
HBsAg detection of NWFET sensors with nanowires of various lengths. *I*_*D*_–*V*_*G*_ curves of HBsAg measured using NWFET sensors with (**a**) 1.6, (**b**) 3, and (**c**) 5 µm nanowires. (**d**) Normalized voltage shifts of NWFET sensors (n = 3).

Figure 8 presents the HBsAg detection results of the NWFET sensors with nanowires of various lengths. The number of nanowires in a transistor was consistently 20. Figure 8a–c presents the *I*_*D*_–*V*_*G*_ curves of HBsAg at various concentrations measured using NWFET sensors with 1.6, 3, and 5 µm long nanowires, respectively. It is noted that the current levels in Figure 8a–c are not the same because the ON current of 1.6, 3, and 5 um-length NWFETs are very different. An increase in the HBsAg concentration caused a rightward shift of the *I*_*D*_–*V*_*G*_ curve. Thus, the proposed detection system used BTP with a pH of 7 as the buffer, and the HBsAg with an isoelectric point of 4.6 carried negative charges. According to the theory of field effect transistor, when the charged biomolecules bind to the nanowire, a large number of negative charges on *V*_*G*_ exert a negative electric field, and causes the *I*_*D*_–*V*_*G*_ curve to shift right. Thus, the results are consistent with this theory. The shift of the *I*_*D*_–*V*_*G*_ curve of the NWFET with a nanowire of 3 µm was larger than that in the curves of the other two types of sensors (Figure 8b). Thus, the concentration was changed from 80 ag/mL to 8 pg/mL. The results revealed that at 80 fg/mL, the shift of the curve was minor. The NWFET sensors with 1.6 and 5 µm nanowires exhibited linear variation in the range of experimental concentrations (800 ag/mL to 800 pg/mL). Figure 8d presents the normalized voltage shifts (n = 3) obtained using the measured results. The concentration-related voltage shifts were linearly fitted. The slope of the straight line represents the sensitivity of the sensor. The intersection of the straight line and the X axis represents the sensor’s limit of detection (LOD). The NWFET biosensors with 1.6 and 5 µm nanowires exhibited an excellent linear range from 800 ag/mL to 800 pg/mL. The slope of the sensors with 3 µm nanowires was divided into three sections. When the concentration was lower than 800 ag/mL, the sensitivity was high. From 800 ag/mL to 800 fg/mL, the sensitivity decreased. When the concentration exceeded 800 fg/mL, the variation in voltage was negligible, indicating that the sensor approached saturation. Table 1 presents the HBsAg detection results of the NWFET sensors with nanowires of various lengths. The NWFET biosensors with 1.6 and 5 µm nanowires exhibited favorable linear detection ranges. The sensors with 1.6 µm nanowires had higher sensitivity than did those with 5 µm nanowires. The sensitivity of the biosensor with 3 µm nanowires was 0.203/decade at low concentrations, 0.061/decade at moderate concentrations (800 ag/mL to 800 fg/mL; identical to that of the sensors with 1.6 µm nanowires), and saturated at 800 fg/mL and higher. The sensor with 3 µm nanowires had the ideal LOD: 4.02 × 10^−18^ g/mL.

**Figure 9:**
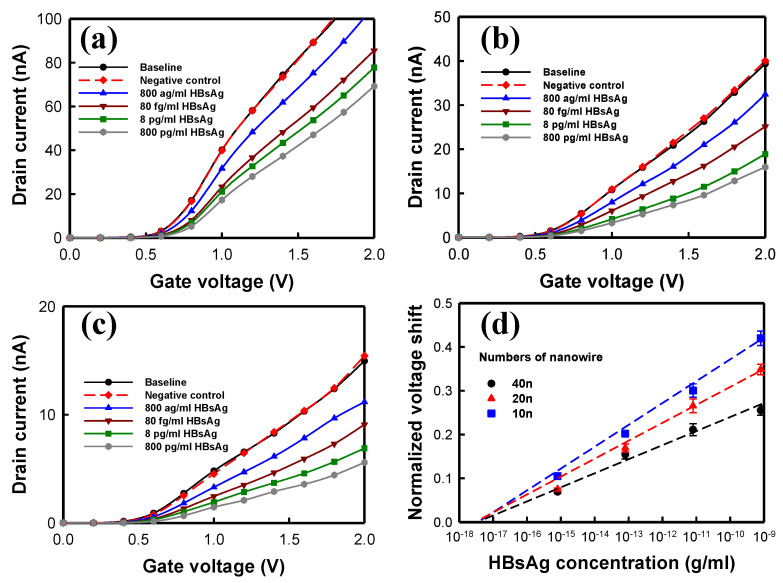
HBsAg detection of the NWFET sensors with different numbers of nanowires. HBsAg *I*_*D*_–*V*_*G*_ curves measured using NWFET sensors with (**a**) 40, (**b**) 20, and (**c**) 10 nanowires. (**d**) Normalized voltage shifts of NWFET sensors.

Figure 9 presents the HBsAg detection of the NWFET sensors with different numbers of 5 µm nanowires. Figure 9a–c presents the *I*_*D*_–*V*_*G*_ curves under various concentrations of HBsAg measured using NWFET sensors with 10, 20, and 40 nanowires, respectively. The detection concentration ranged from 800 ag/mL to 800 pg/mL. As the HBsAg concentration increased, the curves of the three transistors linearly shifted rightward. Figure 9d presents the normalized voltage shifts (n = 3) acquired based on the measured results. The variation in sensor voltage was linearly related to the logarithm of HBsAg concentration, whereas the number of nanowires was inversely proportional to sensor sensitivity. The NWFET sensors with 10 nanowires had higher sensitivity than did the other two sensors. Table 2 presents the HBsAg detection of the NWFET sensors with different numbers of nanowires. The NWFET sensors with 10 nanowires exhibited the highest sensitivity, 0.052/decade. The linear detection ranges of the three sensors were identical, and the difference in their LODs was negligible, all close to the concentration range of approximately 10^−18^ g/mL.

The experimental results regarding the basic electrical properties of the NWFET sensors indicate that short nanowires result in low SS. Theoretically, low SS in transistors causes high sensor sensitivity. However, the results of the HBsAg detection test are inconsistent with this theory. The sensitivity of the sensor with 3 µm nanowires was highest at low concentrations. Because the detection mechanism of nanowire sensors is related to target biomolecules and probe collision probabilities, we inferred that the length of the nanowires affected the number of antibodies on the nanowires and the probabilities of collision between HBsAg and the nanowires. Although the basic electrical properties of the NWFET sensor with 3 µm nanowires indicated inferior performance to that of the sensor with 1.6 µm nanowires, the high collision probability increased the sensitivity of the NWFET sensor with 3 µm nanowires to low concentrations of the biomolecules. Detection at higher concentrations yielded identical results for the sensors with 1.6 and 3 µm nanowires. Collision probability is related to the size of the space, and the size of target biomolecules may affect sensor sensitivity; thus, the detection of protein and biomolecules such as DNA or bacteria may yield different results. The number of nanowires had little effect on the basic electrical properties of the NWFET sensors. However, in the detection of HBsAg, sensors with few nanowires had high sensitivity, which may have been caused by the strengthened effect of HBsAg on the sensors, resulting in considerable variation in the current.

## 4. Conclusions

We used sidewall spacer etching to fabricate poly-Si NWFET biosensors with 100 nm–wide nanowires. The poly-Si NWFET biosensors exhibited favorable electrical properties and high stability in solutions. We developed NWFETs of various sizes and used HBsAg as the detection target to explore the effect of the length and number of nanowires on detection. The experimental results revealed that the *I*_*D*_–*V*_*G*_ curves of the transistors shifted rightward as HBsAg concentration increased. The NWFET sensor with 3 µm nanowires had high sensitivity under low concentrations of biomolecules and sensitivity identical to that of the NWFET with 1.6 µm nanowires under moderate concentrations. When the concentration exceeded 800 fg/mL, the sensitivity reached saturation. The NWFET sensors with 1.6 and 5 µm nanowires exhibited linear variation between concentrations of 800 ag/mL and 800 pg/mL. We also demonstrated that the lower the number of nanowires was, the higher the sensitivity of the NWFET sensor was. Both the length and number of nanowires had little effect on LOD. The results of this study may contribute to the fabrication of NWFET sensors in the future.

## Figures and Tables

**Table 1:** HBsAg detection of NWFET sensors with nanowires of various lengths.

Length of Nanowire	Sensitivity	Linear Range	LOD (g/mL)
1.6 µm	0.061	800 ag/mL to 800 pg/mL	4.69 × 10^−18^
3 µm	0.061 (800 ag/mL to 800 fg/mL) 0.203 (below 800 ag/mL)	800 ag/mL to 800 fg/mL	4.02 × 10^−18^
5 µm	0.046	800 ag/mL to 800 pg/mL	6.69 × 10^−18^

**Table 2:** HBsAg detection of NWFET sensors with different numbers of nanowires.

Numbers of Nanowire	Sensitivity	Linear Range	LOD (g/mL)
40 n	0.030	800 ag/mL to 800 pg/mL	6.85 × 10^−18^
20 n	0.046	800 ag/mL to 800 pg/mL	6.69 × 10^−18^
10 n	0.052	800 ag/mL to 800 pg/mL	5.21 × 10^−18^
